# Chlorhexidine and gauze and tape dressings for central venous catheters: a randomized clinical trial^1^

**DOI:** 10.1590/0104-1169.3443.2478

**Published:** 2014

**Authors:** Edivane Pedrolo, Mitzy Tannia Reichembach Danski, Stela Adami Vayego

**Affiliations:** 2 Doctoral student, Universidade Federal do Paraná, Curitiba, PR, Brazil. Professor, Instituto Federal do Paraná, Curitiba, PR, Brazil; 3 PhD, Adjunct Professor, Universidade Federal do Paraná, Curitiba, PR, Brazil

**Keywords:** Clinical Trial, Chlorhexidine, Catheterization, Central Venous, Technology, Nursing, Bandages

## Abstract

**OBJECTIVE:**

to assess the effectiveness of the chlorhexidine antimicrobial dressing in comparison to the gauze and tape dressing in the use of central venous catheters.

**METHOD:**

a randomized clinical trial was conducted in the intensive care and adult semi intensive care units of a university hospital in the south of Brazil. The subjects were patients using short-term central venous catheters, randomly assigned to the intervention (chlorhexidine antimicrobial dressing) or control (gauze and micro porous tape) groups.

**RESULTS:**

a total of 85 patients were included: 43 in the intervention group and 42 in the control group. No statistically significant differences were found between dressings in regard to the occurrence of: primary bloodstream infections (p-value = 0.5170); local reactions to the dressing (p-value = 0.3774); and dressing fixation (p-value = 0.2739).

**CONCLUSION:**

both technologies are effective in covering central venous catheters in regard to the investigated variables and can be used for this purpose. Registry ECR: RBR-7b5ycz.

## Introduction

The Central Venous Catheter (CVC) is a device largely used by patients in critical health conditions. Its use, however, may entail many complications, among which is Primary Bloodstream Infection (PBSI), which is the most common bloodstream infection in patients with a CVC for more than 48 hours and that is not related to another site^(^ 1 ^)^.

A concern to promote measures that reduce PBSI is justified given the high rates of associated morbidity and mortality. A patient affected by this complication will remain hospitalized for another seven to 21 days, with a consequent increase in hospital costs from U$3,700 to U$29,000 and in a risk of death; attributable mortality for this infection is 18%^(^ 2 ^)^.

In Brazil, it is estimated that 60% of infections are catheter-related PBSI, especially short-term catheters^(^ 3 ^)^. Considering the relevance of this figure, the Brazilian Health Surveillance Agency (ANVISA) has made efforts to prevent this complication. Among the measures adopted is the goal to reduce PBSI rates by 30% in three years^(^ 4 ^)^. Various facilities in the international context have reported the adoption of severe measures to reduce PBSI rates to zero. Given the impact of this complication for patients and healthcare services, a continuous search for effective prevention is justified^(^ 5 ^)^.

Despite the large number of PBSI risk factors, these are minimized with the adoption of interventions upon catheter insertion and maintenance such as: implementation of bundle care (hand hygiene, maximum barrier precautions upon insertion, chlorhexidine skin antisepsis, optimal catheter site, and prompt removal)^(^ 6 ^)^; maintaining the output opening, occluded with sterile dressing; among other interventions^(^ 1 ^,^ 3 ^)^.

In regard to sterile occlusive dressings, there are different technologies on the market, such as gauze and tape, transparent polyurethane film dressing, and chlorhexidine antimicrobial dressing. These vary in terms of durability, ease of application, skin reaction, and ability to prevent infections. Gauze and tape dressings and transparent polyurethane film have been widely investigated recently and meta analysis addressing this topic reports that the latter leads to four times as many infections^(^ 7 ^)^.

Knowledge is still incipient in regard to the chlorhexidine antimicrobial dressing. There are few studies in the international literature, which hinders the incorporation of this new technology into nursing practice. Evidence currently available does not show, with any statistical significance, how effective this technology is in reducing PBSI rates. Hence, both the Center for Disease Control and Prevention and ANVISA recommend the use of this technology only in units where high PBSI rates remain even after adopting all measures recommended by the literature. Its indication for other patients remains elusive^(^ 1 ^,^ 4 ^)^.

Therefore, this study's aim was to assess the effectiveness of chlorhexidine antimicrobial dressings in comparison to gauze and tape dressings for the following outcomes: prevention of primary bloodstream infections, local reactions, and fixation of dressings.

## Method

The study was approved by the Institutional Review Board under the record CEP/SD 1145.070.11.06 and CAAE 0067.0.091.208-11, and registered in the Brazilian Clinical Trials Registry under No. ECR: RBR-7b5ycz. This randomized clinical trial^(^ 8 ^)^ was conducted in the Intensive Care Unit (ICU) and Adult Semi Intensive Care Unit (ASICU) of a university hospital in the South of Brazil from October 2011 to May 2012. The study's participants were severe clinical and surgical patients. 

**Figure 1:**
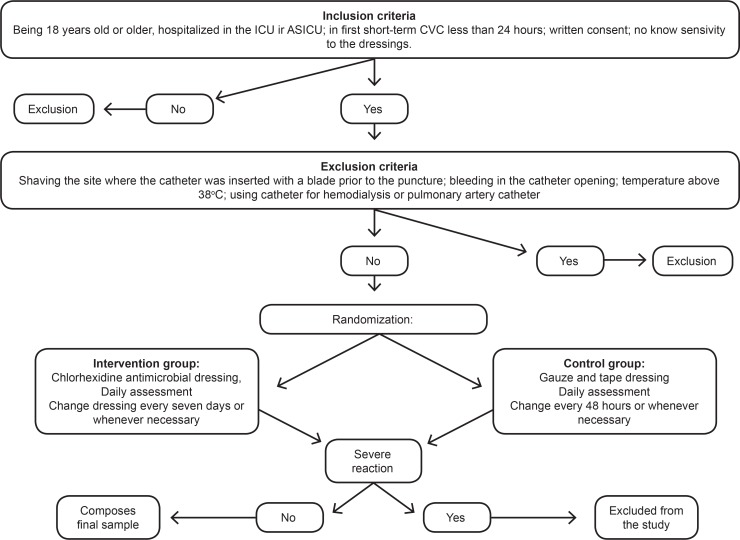
- Flowchart for the inclusion of participants in the study

A pilot test with eight patients was conducted from October 18^th^ to November 2^nd^ to estimate the proportion of CVC-related infections or local reactions. The patients enrolled in this test were not included in the final study. The inclusion of patients in the study followed the scheme shown in Figure 1. We employed a randomized technique in blocks of six patients with a sequence of random numbers generated by Excel^(r)(8)^.

After randomization, the dressings were changed for the first time and the technology to which the patient was assigned was applied. At this point, we collected the socio-demographic and clinical variables, along with catheter data using an instrument defined in the pilot test. Afterwards, the participants were assessed for the collection of the outcome variables as described below.

The outcome variables included: PBSI, fixation of dressing to skin, and local reaction to dressing. In this study, PBSI was the presence of at least one of the following signs and symptoms: fever (>38°); shivering; urine output<20ml/h; systolic blood pressure ≤ 90mmHg; no apparent infection in another site; and medical prescription of antimicrobial therapy for sepsis^(^ 4 ^)^. 

Note that the patients using antimicrobial agents prior to the catheter placement were included in the study provided they did not present clinical diagnosis of sepsis; the PBSI criteria established in the literature did not indicate the need to exclude these patients^(^ 4 ^)^.

The following information, collected daily, was standardized to establish the infection variable: temperature>38ºC, systolic blood pressure<90 mmHg, oliguria<20ml/h, tenderness, pain or swelling on palpation, hyperemia, cyanosis or discharge at the catheter opening. In the event of a clinical suspicion of PBSI, a blood culture or a culture of the catheter tip was collected according to medical recommendations, a routine procedure of the service where the study was conducted. The catheter tip was collected only for patients with sepsis for whom it was not possible to define the infectious focus by blood culture only.

For the local reaction variable, we observed signs and symptoms. Local reaction was considered to be present when the patient presented at least one of the following: maceration, hyperemia, desquamation or itching in the region where there was contact between skin and dressing. For the fixation variable, we assessed whether the edges were detached, regardless of whether the integrity of the dressing was compromised. Good fixation was considered to have occurred when the dressing remained intact in more than 75% of the observations. The analysis of variables, local reaction and fixation was performed always at the same time by a staff member previously trained for data collection and, if there was doubt, a second evaluator would assess the variables, as well.

The chlorhexidine antimicrobial dressing was changed every seven days and gauze and tape every 48 hours^(^ 2 ^)^, or less whenever the dressing edges detached and compromised the dressing integrity or discharge accumulated in the catheter opening^(^ 2 ^)^. For the catheter puncture, we used the standardized technique adopted by the unit that is based on the care bundle recommended by international clinical guidelines^(^ 1 ^,^ 6 ^)^. A qualified staff member exclusively performed the dressings using a standardized technique. Patients were monitored up to the end point, which comprised transference to another hospital facility, puncture of a second catheter, or its removal. Patients discharged from the ICU or ASICU to a hospitalization ward were also monitored up to the aforementioned end points.

A proportion of PBSI of 60% was considered for the control group for the sampling computation based on the pilot test. A sample of 42 patients was estimated for each group (power of 0.80 (1 - β = 0.80)), with a minimum difference of 30% between treatments and levels of significance of 0.05 (α = 0.05). Data were analyzed using descriptive statistics associated with Fisher's exact test and Williams' G-test (qualitative variables) and the Mann-Whitney U-test (quantitative variables), considering a level of significance of 5%.

## Results

A total of 43 patients were included in the intervention group (chlorhexidine antimicrobial dressing - CHD) and 42 in the control group (gauze and tape dressing). There was no follow-up loss of the patients included in the study. Socio-demographic and clinical data are presented in Table 1 and catheter-related data are presented in Table 2. The average age of the participants in the CHD group was 55.1 (SD=16.3) years old and that of the participants in the gauze and tape group was 60.2 (SD=18.9) years old, with no statistical difference (p = 0.0929). All the analyzed catheters were made of polyurethane.

**Table 1:** - Distribution of frequency (n) and percentage (%) of socio-demographic and clinical variables of patients in the intervention (chlorhexidine antimicrobial dressing) and control (gauze and tape) groups. Curitiba, PR, Brazil, 2012

Variable		Chlorhexidine Intervention group (n=43)			Control group dressing (n=42)		p-value
		n	%		n	%	
Sex							0.5379*
	Female	19	44.2		18	42.9	
	Male	24	55.8		24	57.1	
Ethnicity							0.1663*
	Caucasian	36	83.7		39	92.9	
	African-descendant	7	16.3		3	7.1	
Primary focus of pathology							0.2669^†^
	Digestive system	18	41.9		9	21.4	
	Nervous system	10	23.2		11	26.2	
	Malignant neoplasia	7	16.3		7	16.7	
	Circulatory system	2	4.6		5	11.9	
	Other	6	14.0		10	23.8	
Comorbidities							0.2683^†^
	One	5	11.6		12	28.6	
	Two	13	30.2		8	19.1	
	Three	5	11.6		4	9.5	
	Four or more	2	4.7		4	9.5	
	No comorbidities	18	41.9		14	33.3	
Hospitalization unit							0.2298*
	Intensive Care Unit	40	93.0		36	85.7	
	Semi-intensive care unit	3	7.0		6	14.3	
Discharge or death							0.3784*
	Discharge	27	62.8		24	57.1	
	Death	16	37.2		18	42.9	
Antimicrobial before puncture							0.5152^†^
	One antimicrobial	6	14.0		10	23.8	
	Two antimicrobial	9	20.9		8	19.1	
	Three or more antimicrobial	3	7.0		5	11.9	
	No	25	58.1		19	45.2	

* Fisher's exact test

† G-test

**Table 2:** - Distribution of frequency (n) and percentage (%) of catheter-related variables in patients within the intervention (chlorhexidine intervention group) and control (gauze and tape dressing) groups. Curitiba. PR, Brazil, 2012

Variable		Chlorhexidine Intervention group (n=43)			Control group dressing (n=42)		p-value
		n	%		n	%	
Indication							0.8053*
	Administration of vasoactive drugs	38	88.4		37	88.1	
	Monitoring	4	9.3		3	7.1	
	Total parenteral nutrition	1	2.3		2	4.8	
Unit where puncture was performed							0.9654*
	Clinical	1	2.3		1	2.4	
	Surgical center	17	39.6		17	40.5	
	Semi-intensive care unit	5	11.6		7	16.7	
	Intensive care unit	16	37.2		13	30.9	
	Emergency room	4	9.3		4	9.5	
Number of lumen catheter							0.6156^†^
	One	5	11.6		5	11.9	
	Two	38	88.4		37	88.1	
Anatomical site of catheter insertion							0.2948^†^
	Subclavian	31	72.1		27	64.3	
	Jugular	12	27.9		15	35.7	
Reason follow-up ceased							0.9678*
	Catheter removal	25	58.2		23	54.8	
	Death	11	25.6		11	26.2	
	Placement of a second catheter	5	11.6		5	11.9	
	Non-infectious diseases	2	4.6		3	7.1	

* G-test

† Fisher's exact test

The average time of hospitalization was similar between groups: 9.7 (SD=13.7) days for the CHD group and 9.5 (SD=9.1) for the gauze and tape group (p = 0.1418). Duration of catheter placement was 4.9 (SD=2.5) days for the CHD group and 5 (SD=2.7) days for the gauze and tape group, with no statistical difference (p = 0.1418).

No significant differences were found between the dressings in regard to the occurrence of PBSI (p-value = 0.5170). Laboratorial confirmation of PBSI was obtained through blood cultures in 16 patients from the chlorhexidine group (37.21%) and in 17 patients from the gauze and tape group (40.48%), and with analysis of the catheter tip in three patients from the chlorhexidine (6.98%) group, for whom establishment of infectious focus was not possible through blood culture only. Four clinical PBSI and one with laboratorial confirmation, in which the *Candida krusei* microorganism was isolated, were observed in the gauze and tape group. All the cases in the CHD group were clinical (Table 3).

**Table 3:** - Variables related to primary bloodstream infections, local reactions and fixation of dressing in patients from the intervention (chlorhexidine antimicrobial dressing) and control (gauze and tape) groups. Curitiba, PR, Brazil, 2012

Variable		Chlorhexidine Intervention group (n=43)			Control group dressing (n=42)		p-value
		n	%		n	%	
Primary bloodstream infection		6	13.95		5	11.90	0.5170*
Local reaction to dressing		17	39.53		19	45.24	0.3774*
Signs and symptoms of local reaction							
	Hyperemia	9	20.93		9	21.43	0.5824*
	Maceration	10	23.26		12	28.57	0.3777*
	Desquamation	4	9.30		2	4.76	0.3493*
	Itching	3	6.98		3	7.14	0.6507*
Fixation							0.2739*
	Good	36	83.72		38	90.48	
	Poor	7	16.28		4	9.52	

* Fisher's exact test

Association between signs and symptoms at the catheter opening and the occurrence of PBSI was tested regardless of the type of dressing used and no statistical significance was found between PBSI and the variables: hyperemia at the opening (p-value = 0.2042), cyanosis at the opening (p-value= 0.6181), serous discharge (p-value = 0.4255), serosanguineous discharge (p-value = 0.5881), sanguineous discharge (p-value = 0.1048), purulent discharge (p-value = 0.3420) swelling upon palpation (p-value = 0.2993), and pain to palpation (p-value = 0.2695).

Both dressings presented good fixation and the participants presented a high incidence of local reaction (39.53% - chlorhexidine and 45.24% - gauze) with no statistically significant differences between groups. Most cases of local reaction were characterized by skin hyperemia and maceration. Note that there was one case of a severe reaction to the dressing in the intervention group and for this reason that participant was excluded from the study (Table 3).

## Discussion

Catheter-related infection is a complication that greatly influences the morbidity and mortality of patients using CVCs, a fact that justifies unceasing efforts to promote preventive evidence-based interventions. The technologies addressed in this study are safe options for occluding catheter openings due to the low incidence of PBSI in both groups (13.95% - CHD; 11.90% - gauze). No significant differences were found between the dressings in regard to the occurrence of PBSI (p-value = 0.5170). The incidence of PBSI, however, can be even lower, as shown in a cohort study conducted in seven ICUs that reported an infection rate 6.4%^(^ 9 ^)^.

One meta analysis and two clinical trials^(^ 11 ^-^ 12 ^)^ were found in an extensive search of the literature^(^ 10 ^)^. These studies assessed the ability of chlorhexidine dressings to reduce colonization rates and PBSI. Nonetheless, all studies used transparent polyurethane dressings in the control group. This is the first Brazilian study addressing this new technology that also employs gauze and tape as the control group. Note that this is a safe choice, since the meta analysis included six clinical trials comparing gauze and tape with transparent polyurethane and revealed no differences between these technologies in regard to the incidence of catheter-related infectious complications^(^ 7 ^)^.

A meta analysis that included eight randomized clinical trials assessing chlorhexidine-impregnated dressing showed that this technology has a tendency to reduce PBSI, though with no statistical evidence^(^ 10 ^)^. Two new clinical trials were published in 2009 addressing this topic and both showed significant decrease in PBSI among patients using chlorhexidine dressings^(^ 11 ^-^ 12 ^)^. The studies, however, used a sponge impregnated with chlorhexidine, which needs to be covered with transparent polyurethane dressing. In this study, the chlorhexidine dressing used consists of a transparent film, the adhesive of which contains chlorhexidine gluconate gel at 2%.

The reduced indexes of PBSI in ICU are a result of the massive adoption of preventive measures on the part of the medical and nursing staffs. Additionally, constant monitoring of patients is an important factor that culminates in the removal of the device and early identification of complications. The adoption of these measures in the studied units may have limited the results found, especially in regard to the early removal of devices.

Note that the absence of statistically significant differences in the effectiveness of chlorhexidine and gauze and tape dressings in the prevention of PBSI may have been influenced by the fact that only the first CVC of each patient was included in the analysis, since the more frequently patients are exposed to CVC, the higher the risk of complications. Another important factor is related to the short duration of catheter placement, as periods longer that five days are associated with a higher risk of infection.

The PBSI-related microorganisms most frequently found are *Staphylococcus* coagulase-negative, *Staphylococcus aureus, Enterococcus *and* Candida* spp.^(^ 1 ^)^. The only case of infection confirmed by a laboratory was associated with the *Candida krusei *microorganism.

Hyperemia (20.93% - CHD; 23.81% - gauze) in the catheter opening was the sign most frequently observed in this study, while purulent discharge was the least observed (4.65% - CHD; 2.38% - gauze), though none presented significant association with the occurrence of PBSI. One study conducted with 37 patients who acquired PBSI in a university hospital found the following to be the two most frequent signs: purulent discharge (27%) and hyperemia in the catheter opening (18.9%)^(^ 13 ^)^. International clinical guidelines recommend daily inspection and palpation of the catheter site to check for signs of inflammation ^(^ 1 ^)^. Data resulting from palpation, however, such as edema (p-value = 0.2993) and pain (p-value = 0.2695) did not present a significant relationship with the PBSI outcome.

The dressings analyzed in this study were associated with local reactions (CHD - 39.53%; gauze - 45.24%), however, no statistical significant difference was found between the groups. According to the data found, one randomized clinical trial conducted with 21 ICU inpatients also reported a high rate of gauze and tape-related local reaction (60%)^(^ 14 ^)^. The clinical signs more frequently observed for gauze and tape dressings were skin maceration (28.57%) and hyperemia (21.43%). Even though the micro-porous tape is hypoallergenic, the signs observed were concentrated in the region in which skin was in contact with the tape and not in the region of direct contact between the gauze and the patient's skin.

In regard to the chlorhexidine dressing, the part composed of transparent film is hypoallergenic, however chlorhexidine is associated with hypersensitivity. The clinical signs most frequently observed in patients using this type of dressing were skin maceration (28.57%) and hyperemia (21.43%), which were concentrated in the entire region of contact between the dressing and skin. This variable was assessed because a local reaction may compromise the integrity of peri-catheter skin and increase the likelihood of greater colonization in the region.

There was one case of a severe reaction to CHD, a fact that caused discontinuing the patient in the study. We note, however, the low incidence of severe reactions to the dressing, a fact that would impede its widespread use and reaffirms the view that this dressing should be prescribed only for patients who do not present known hypersensitivity to chlorhexidine antisepsis. There is one study corroborating these findings, as it reports that chlorhexidine is well-tolerated by patients^ (15)^.

The technique used to apply dressings to cover CVCs is sterile with the use of material free of microorganisms that may cause infection due to continued solution in the region of the catheter opening area. In order to maintain low bacterial load in this region, the dressing must strongly adhere to skin to prevent the catheter opening from coming into contact with air. Note that both studied groups presented good fixation (83.72% - CHD; 90.48% - gauze); no statistical differences were found in regard to dressing fixation (0.2739).

Two studies assessed the variable for the fixation of dressing, one of which assessed gauze and tape fixation and the other assessed the fixation of the chlorhexidine dressing. The randomized clinical trial assessing chlorhexidine dressing included 1,636 patients. It reports that approximately 40% of the dressing changes were detached from the skin, which led to early changing of the dressing^(^ 11 ^)^. The randomized clinical trial assessing gauze and tape dressing shows that 50% of the dressings used in the analyzed catheters presented poor fixation and required early changing^(^ 14 ^)^. In this study, the rates of poor fixation found for both the gauze and tape and chlorhexidine dressings were lower than that reported in the literature. Note that the good fixation of dressings found in this study is a factor relevant for the maintenance of occluded dressings, which favors the reduced colonization of peri-catheter skin.

Limitations of this study include the fact that the severity of the patients' clinical conditions was not related to the investigated outcomes, nor were the nutritional states of patients. These are factors that directly impact the susceptibility of patients to infectious complications and likely factors linked to the PBSI outcome. Additionally, patients using antimicrobial agents may have masked the occurrence of sepsis with the action of medications, a fact that may have interfered in the studied outcome and, therefore, is a limitation of this study.

## Conclusion

The objective was to assess the effectiveness of the chlorhexidine antimicrobial dressing in comparison to the gauze and tape dressing for the outcomes: prevention of primary bloodstream infections, local reactions, and the fixation of dressings.

This study shows that, in a unit that adopts catheter bundle care, the chlorhexidine antimicrobial dressing is not effective in reducing PBSI when compared to the gauze and tape dressing. In regard to the occurrence of local reactions and the fixation of dressings, no statistically significant differences were found between groups. A high rate of local reaction was observed in both groups, as well as the occurrence of a severe skin reaction in the case of one patient using the chlorhexidine dressing.

Both the technologies addressed in this study are effective in covering CVCs in regard to the assessed outcomes and can be employed for this purpose. We recommend further research to consolidate scientific evidence for the use of chlorhexidine dressing in the prevention of primary bloodstream infections in critical patients.

## References

[1] O'Grady NP, Alexander M, Burns LA, Dellinger EP, Garland J, Heard SO (2011). Guidelines for the prevention of intravascular catheter-related
infections.

[2] Springhouse (2010). As melhores práticas de enfermagem: procedimentos baseados em
evidências.

[3] Agência Nacional de Vigilância Sanitária (BR) (2010). Indicadores nacionais de infecções relacionadas à assistência a
saúde - corrente sanguínea.

[4] Agência Nacional de Vigilância Sanitária (BR) (2010). Orientações para prevenção de infecção primária de corrente
sanguínea.

[5] Southworth SL, Henman LJ, Kinder LA, Sell JL (2012). The journey to zero central catheter−associated
bloodstream infections: culture change in an intensive care unit. Crit Care Nurse..

[6] Osorio J, Álvarez D, Pacheco R, Gómez CA, Lozano A (2013). Implementation of an insertion bundle for preventing
central line-associated bloodstream infections in an Intensive Care Unit in
Colombia. Rev Chil Infectol..

[7] Webster J, Gillies D, O'Riordan E, Sherriff KL, Rickard CM, The Cochrane Library (2011). Gauze and tape and transparent polyurethane dressings
for central venous catheters (Cochrane Review). http://onlinelibrary.wiley.com/doi/10.1002/14651858.CD003827.pub2/abstract.

[8] Hulley SB, Cummins SR, Browner WS, Grady DG, Newman TB (2008). Delineando a pesquisa clínica: uma abordagem
epidemiológica.

[9] Mesiano ERAB, Merchán-Hamann E (2007). Bloodstream infections among patients using central
venous catheters in intensive care units. Rev. Latino-Am. Enfermagem..

[10] Ho KM, Litton E (2006). Use of chlorhexidine-impregnated dressing to prevent
vascular and epidural catheter colonization and infection: a
meta-analysis. J Antimicrob Chemother..

[11] Timsit JF, Schwebel C, Bouadma L, Geffroy A, Garrouste-Orgeas M, Pease S (2009). Chlorhexidine-impregnated sponges and less frequent
dressing changes for prevention of catheter-related infections in critically ill
adults: a randomized controlled trial. JAMA..

[12] Ruschulte H, Franke M, Gastmeier P, Zenz S, Mahr KH, Buchholz S (2009). Prevention of central venous catheter related infections
with chlorhexidine gluconate impregnated wound dressings: a randomized controlled
trial. Ann Hematol..

[13] Marques Netto S, Echer IC, Kuplich NM, Kuchenbecker R, Kessler F (2009). Infecção de cateter vascular central em pacientes
adultos de um centro de terapia intensiva. Rev Gaúcha Enferm..

[14] Pedrolo E, Danski MTR, Mingorance P, De Lazzari LSM, Johann DA (2011). Clinical controlled trial on central venous catheter
dressings. Acta Paul Enferm..

[15] Frasca D, Dahyot-Fizelier C, Mimoz O (2010). Prevention of central venous catheter-related infection
in the intensive care unit. Critical Care..

